# 1502. Disseminated *Mycobacterium-avium* complex in People with HIV in México City: a Single-center, 23 year (1999-2022) Retrospective Cohort Study.

**DOI:** 10.1093/ofid/ofad500.1337

**Published:** 2023-11-27

**Authors:** Alejandro Ortiz Hernández, Lorena Guerrero-Torres, Rodrigo Ville Benavides, Rafael Valdes Ventura, Yanink-Nereid Caro-Vega, Brenda-Eloisa Crabtree-Ramírez

**Affiliations:** Instituto Nacional de Ciencias Médicas y Nutrición "Salvador Zubirán", Tlalpan, Distrito Federal, Mexico; Instituto Nacional de Ciencias Médicas y Nutrición "Salvador Zubirán", Tlalpan, Distrito Federal, Mexico; Instituto Nacional de Ciencias Médicas y Nutrición "Salvador Zubirán", Tlalpan, Distrito Federal, Mexico; Instituto Nacional de Ciencias Médicas y Nutrición "Salvador Zubirán", Tlalpan, Distrito Federal, Mexico; Instituto Nacional de Ciencias Médicas y Nutrición "Salvador Zubirán", Tlalpan, Distrito Federal, Mexico; Instituto Nacional de Ciencias Médicas y Nutrición "Salvador Zubirán", Tlalpan, Distrito Federal, Mexico

## Abstract

**Background:**

Despite universal access to combined antiretroviral therapy (ART) in Mexico since 2005, people with HIV (PWH) still present with late-stage complications due to delayed diagnosis and/or entry into care. Among these complications, disseminated *Mycobacterium-avium* complex infection (DMAC) remains a concerning issue given its high mortality and diagnostic challenges. Therefore, we aimed to describe the clinical and microbiological characteristics and outcomes of PWH with DMAC in a tertiary care center in Mexico City over a period of 23 years.

**Methods:**

In this retrospective study, we reviewed all records of PWH attending our hospital from 1999-2022. We included adult PWH with any positive extra-pulmonary MAC-culture. We developed a standardized case report form and recorded sociodemographic, clinical, and microbiological characteristics. We followed patients until microbiological cure or death.

**Results:**

We included 37 PWH diagnosed with DMAC infection, of whom 34 (92%) were men. Overall, 4 (11%) were diagnosed with DMAC before 2005 and 33 (89%) after 2005. The median age was 34 years (IQR 27-36 years). Following HIV diagnosis, mean time to diagnosis of DMAC was 14.2 months (IQR 1.6-11.3 months). At DMAC diagnosis, 12 (32%) patients were on ART and 25 (68%) were ART naïve. Fever was the most common presenting symptom, affecting 32 patients (86%). Mean time to microbiological diagnosis was 34 days. The most common sites for culture isolation were blood (76%) and bone marrow (70%). Anemia occurred in 34 patients (92%), followed by leukopenia in 23 (62%) and pancytopenia in 9 (24%). All patients required hospitalization, with 32 (86%) receiving DMAC-specific treatment and 5 (14%) dying before treatment initiation. Thirty-four patients (92%) received a clarithromycin-based regimen, 15 (41%) of patients developed IRIS. Overall mortality during follow-up was 51%, with 79% deaths occurring within the first year after DMAC diagnosis.

**Conclusion:**

Despite universal ART access, DMAC remains a high-mortality AIDS-defining illness in Mexico City. Efforts must be made to promote HIV testing, early diagnosis, and entry into care.

**Disclosures:**

**All Authors**: No reported disclosures

